# Medical Men and the Workmen's Compensation Act

**Published:** 1909-09-25

**Authors:** 


### Medico-Legal Points.

### MEDICAL MEN AND THE WORKMEN'S COMPENSATION ACT.

From a study of numerous compensation cases which have recently been heard by County Court judges and in the Court of Appeal, the function of the medical man is daily becoming more and more important. This is largely due to the changes in the law which were made by the Workmen's Compensation Act of 1906. Employers are now liable if their workmen become affected by certain industrial diseases. Claims are made in respect of other diseases alleged to have been brought on or accelerated by "accident." Workmen may be compelled in certain cases, to undergo operations or courses of treatment. Lastly, it may sometimes devolve upon a medical man to decide whether an accident has caused a workman "serious and permanent injury." In all these instances the medical evidence is of supreme importance to the administration of justice.

It is proposed in the present article to draw attention to certain recent cases in the County Courts and elsewhere, a record of which may be useful to practitioners who are from time to time consulted with reference to contested cases. It will be convenient to divide the subject as follows: (1) The meaning of an accident; (2) Diseases and diseases following accidents; (3) Compulsory operations; (4) Meaning of serious and permanent injury.

### The Meaning of an "Accident."

(*a*) *Senile Dementia*: It is well known to all medical men who have to do with the Act that the injury to the workman must have been caused by accident arising out of and in the course of the employment. In a case heard at Derby, an engine-driver injured his head and eye by a fall into a pit. This laid him up for six weeks, and he then returned to work. Five years later he was discharged on account of senile dementia. It was sought to connect this condition with the accident, but the County Court judge refused to allow the claim.

(*b*) *Death under Chloroform*: In a case heard in the Court of Appeal on March 26, the applicant had had his hand injured. An operation was performed to avoid the necessity of amputating the hand. A second operation became necessary to prevent contraction. During this operation the man succumbed to the anæsthetic, and his widow claimed compensation for his death. The County Court judge refused the claim on the ground that death was not caused by the accident; but the Court of Appeal allowed the claim on the ground that the operation was part of the treatment reasonably undergone for the purpose of curing the man.

(*c*) *Rupture of a Blood-vessel*: In a case heard at Sheffield, it appeared that a miner, aged 60, suffered dislocation of the shoulder by an accident, which occurred in May 1905. He went to hospital for six weeks, and was then discharged. On September 13, 1908, he died from rupture of a blood-vessel. A medical man, called on behalf of the applicant, who was the widow of the deceased, said that when he saw the applicant just before his death the shoulder was still dislocated, and that the violent movement of the arm sufficient to cause dislocation might cause rupture. A number of other medical witnesses having stated that the death had nothing to do with the accident, the claim was dismissed.

(*d*) *Squinting*: A case which was heard at Wigan illustrates the fact that in order to found a claim for compensation, it must be shown that the accident causes incapacity. In the case in question an injury received by a miner caused him to suffer from a squint. It was alleged that by setting up double vision, this rendered it dangerous for him to follow his calling in the mine. The doctor called on behalf of the applicant, however, admitted that the effect of the accident had not been to diminish the man's working capacity. It was also proved to the satisfaction of the judge that plenty of men with one eye did work in coal mines without any dreadful consequences. Upon these facts the claim was dismissed.

### Diseases and Disease Following Accidents.

In several cases attempts have been made to render employers liable for diseases which followed upon accidents. In some cases the applicants have succeeded; in others the claims have been dismissed. The decision has always been a matter of medical evidence, and in one case at least the judge has placed absolute reliance on the decision of the medical referee.

(*a*) *Cancer*: In a case heard at Newport, a collier sustained a rupture when at work. Five months later he died of cancer. Evidence for the widow tended to show that the cancer was the result of the accident, but for the defence several doctors said the cancer must have been in existence for many months previous to the accident. The judge found for the respondents.

(*b*) *Consumption*: In a case heard at Doncaster, a miner was injured by a blow in the ribs. He was treated for some time. He died of tuberculosis five months after the accident. A medical witness called on behalf of the widow said that there was no trace of consumption four months before the accident. Another doctor expressed the opinion that there had been some latent tubercle present which was stirred up by the accident. A doctor called on behalf of the employers admitted that if a man was perfectly healthy before the accident and died afterwards, he would attribute his death to the accident. Upon these facts the claim was allowed. With this decision we may contrast a case which was decided at Durham. There a lad was injured by a strain in the course of his work on January 31, 1908. He received compensation until the middle of August, and died of consumption in September. It was shown that he developed this complaint some two months after the accident. Upon the medical evidence, however, the judge came to the conclusion that the strain had not caused or accelerated the disease. The employers were therefore excused from liability.

(*c*) *Gas Poisoning*: In a case heard at Droitwich, a claim was made by a woman whose husband was alleged to have been killed by coal-gas poisoning. On November 6, 1908, he was overcome by a rush of gas when tapping a pipe. On the 16th of the same month he complained of giddiness and headache, a sensation of "pins and needles" in his left hand, and that things appeared to be double. There also appeared to be partial paralysis of the muscles of the mouth, and the pupils of his eyes were unequal. He subsequently died. At the trial evidence was tendered to show that the above symptoms were compatible with gas poisoning. The County Court judge held that even if it had been proved that death was due to gas poisoning he could not hold the employers liable, as this was not an industrial disease.

(*d*) *Hemiplegia*: In a case heard at the Ormskirk County Court, the applicant claimed compensation for total incapacity. He had already suffered an accident in which he lost one eye, and in respect of which he received 2s. 6d. a week compensation. It appears that in November last he caught his right knee against the iron bolt of a wagon. Two days later he had an apoplectic seizure from which he was still suffering. According to the medical officer for Wigan, who gave evidence for the applicant, he was suffering from hemiplegia, attributable to the accident; but two other doctors said it was impossible for this condition to have arisen from the accident. The County Court judge awarded four weeks' compensation at 10s. 6d. per week.

(*e*) *Loss of Sensation*: In a case heard at Cardiff, a miner who had met with an accident suffered from complete loss of sensation in one leg. In spite of this the County Court judge held that he was in a fit condition to do ordinary work, and refused to make any award. The medical evidence, however, was to the effect that the applicant's mental condition was such that he could not make the effort to carry out his work. The Court of Appeal held that an award should have been made. The Master of the Rolls said: "It is a fallacy to say that a man loses his right to compensation when the muscular mischief is ended and the nervous, mental and hysterical remains."

(*f*) *Miner's Nystagmus*: In a case heard at Durham it was alleged that the applicant had contracted "miner's nystagmus," which is a recognised industrial disease. The County Court judge referred the matter to Sir Thomas Oliver, as medical referee, who reported that he found no trace of nystagmus. Upon this the learned judge said that, although the man was undoubtedly suffering from something, he must find a verdict for the employers.

(*g*) *Ringworm*: Although it is true that compensation is only paid for recognised industrial diseases, nevertheless, if it can be shown that an ordinary disease was contracted by an accident, the employer may be made liable. The most recent illustration of this is afforded by a case at St. Helens, where a claim was preferred by a youth suffering from ringworm. It was proved that the lad had been sent by his employer, a blacksmith, to attend a horse suffering from this complaint. Upon the medical evidence the County Court judge was satisfied that the applicant had caught the disease from the horse, and he was therefore awarded compensation.

### Compulsory Operations.

In many recent cases applicants for compensation have been ordered either to undergo some operation or to submit to treatment. It is not necessary to refer to the authorities which have laid it down that a County Court judge may refuse compensation if workmen are unreasonable in this regard. There is one point, however, which should be borne in mind. It is that if the medical practitioner who is attending the workman is of opinion that an operation would be dangerous, this will be quite sufficient to justify the refusal. In a recent case, where a number of medical men called by the employers were of opinion that the operation was safe, the Court of Appeal held that the workman was justified in refusing, because of the advice of his own medical man. The Master of the Rolls pointed out that a man could not be unreasonable in following the advice of his own doctor.

### The Meaning of Serious and Permanent Injury.

A question of great importance to medical practitioners arises where the accident which has caused the injury is due to the "serious and wilful misconduct" of the workman. In such cases the employer is only liable if the accident has caused "serious and permanent injury." The existence of this condition must be decided according to the medical evidence. In a recent case at the Stoke County Court, Judge Ruegg was compelled to find that an accident had been caused by the serious and wilful misconduct of the applicant in breaking one of the colliery rules. The question then arose, "Was the injury serious and permanent?" Owing to a conflict of medical testimony, his Honour left this question to a medical referee, who, after careful examination of the workman, answered it in the affirmative. In these circumstances he was bound to make an award in favour of the applicant. As to the meaning of the phrase, his Honour said: "By serious and permanent I do not understand the Act of Parliament to mean that it must necessarily be a lifelong injury, but the injury was such that the effects would not be temporary, and was therefore correctly described as permanent."

